# Role of Side-Chain Length and Counterion Mediation on Dimerization of Rigid Sphere-Rod Amphiphiles: A Molecular Dynamics Investigation

**DOI:** 10.1021/acs.langmuir.5c05734

**Published:** 2026-01-24

**Authors:** Farzad Toiserkani, Yifan Zhou, Abdol Hadi Mokarizadeh, Javad Tamnanloo, Tianbo Liu, Mesfin Tsige

**Affiliations:** School of Polymer Science and Polymer Engineering, 1076 The University of Akron, Akron, Ohio 44325, United States

## Abstract

Rigid sphere-rod amphiphiles (RSRAs) comprising a Keggin polyoxometalate (POM) headgroup and an oligofluorene rod with different side chain lengths (C2, C6, C10, C16, are the number of carbon atoms per side chain) were probed by all-atom molecular dynamics in THF/water mixtures (15 and 33 vol % THF) with tetrabutylammonium (TBA^+^) counterions to elucidate the molecular origins of dimerization. Despite strong Keggin-Keggin electrostatic repulsion, stable dimers form through a synergy of (i) hydrophobic rod/side-chain interaction that strengthens with side-chain length and (ii) counterion-mediated attraction by TBA^+^, which localizes near the Keggins to screen electrostatic repulsion. Packing evolves from near-parallel rods for short chains to interdigitated, tilted arrangements for long chains, while solvent reorganizes cooperatively. Water is depleted from the inter-rod gap, while tetrahydrofuran (THF) accumulates on exterior hydrophobic surfaces as a loose solvation shell. Around Keggins, terminal and bridging oxygens sustain a structured hydration layer with long water residence time. Dynamically, single-molecule root-mean-square deviation (RMSD) increases with side-chain length while dimer self-diffusion decreases modestly. Trends are consistent across solvent fractions, with expected shifts in magnitudes. These results provide an atomistic framework linking side-chain architecture, counterion screening, and solvent organization to the thermodynamic stabilization and dynamic behaviors of RSRA dimers, clarifying early events that preceded higher-order self-assembled structures.

## Graphical abstract

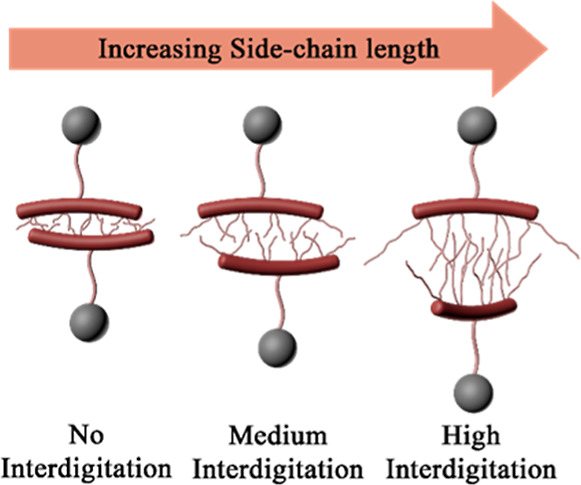

## Introduction

Amphiphilic molecules self-assemble into well-defined nanostructures, a process fundamental to soft matter chemistry and materials science with significant implications for drug delivery,^1,2^ nanotechnology,^3^ and advanced materials development.^4,5^ Various types of amphiphilic molecules, such as surfactants,^6–9^ block copolymers,^10–13^ and dendrimers,^14,15^ contain both hydrophilic and hydrophobic segments. In selective solvents, these molecules self-assemble spontaneously to minimize the system’s free energy, resulting in diverse morphologies such as micelles,^16^ vesicles,^17^ bilayers,^18^ and multilayers structures.^19–29^ The formation of these morphologies is believed to depend on the molecular flexibility of the amphiphiles, enabling them to adjust their chain bending and adopt favorable conformations.

A common feature of previous studies is their focus on fully flexible (i.e., both hydrophobic and hydrophilic blocks are flexible) or semiflexible (i.e., either hydrophobic or hydrophilic segment is rigid) amphiphilic molecules, which provide the capacity for conformation change during the self-assembly process. In contrast, Luo et al.^30^ for the first time demonstrated that a rigid sphere-rod amphiphile composed of a rigid charged Keggin-type cluster (sphere) and a rigid hydrophobic oligofluorene (OF) rod can self-assemble into controllable and responsive onion-like structures with constant interlayer distance through counterion-mediated attraction as well as the rod-to-rod interdigitation rather than chain bending. Their study revealed that assemblies’ size and number of bilayers can be precisely tuned by solvent polarity, ionic strength, temperature, and amphiphile concentration. Based on these observations, Zhou et al.^31^ further examined how solvent polarity regulates the self-assembly of fully rigid sphere-rod amphiphiles, leading to polarity-dependent complex phase transitions. Collectively, these studies revealed the roles of counterion mediation, hydrophobic interaction, and solvent polarity in governing the self-assembly of rod–sphere amphiphiles.

Despite these advances, experimental techniques often face challenges in elucidating the detailed molecular-level mechanisms of self-assembled structures. Limitations in spatial and temporal resolution have hindered a comprehensive understanding of the dynamic processes and interactions that govern their formation. This gap in knowledge has motivated the increasing use of computational simulations as a complementary approach to investigate these complex phenomena at the molecular level.

Computational simulations have advanced our understanding of the self-assembly processes that lead to multilayer structures.^19,21,27,32,33^ To the best of our knowledge, most computational studies on amphiphilic multilayer structure (such as onion-like structures) formation have so far relied on dissipative particle dynamics simulations. Similarly, experimental studies demonstrated that amphiphilic architectures could form multilayered vesicles through tunable parameters, such as temperature, polymer rigidity, concentration, and molecular symmetry, revealing distinct fusion and rearrangement pathways.

The self-assembly of rigid sphere-rod amphiphiles is proven to begin with the formation of dimers, which then merge into larger supramolecular structures.^30^ However, it is difficult to elucidate how counterion mediation, side-chain-length-dependent hydrophobic interactions, and solvation effects collectively govern the initial dimerization process by experimental methods. In particular, the structure of the solvation shell around the amphiphilic molecules plays a crucial role in stabilizing the intermediate, i.e., dimer, in the self-assembly process and influencing both counterion organization and hydration dynamics. Moreover, Zhou et al.,^34^ who synthesized similar rigid amphiphilic molecules, demonstrated that side-chain length plays a crucial role in the self-assembly of rigid sphere-rod amphiphiles. Although coarse-grained simulations have indicated that dimer formation serves as a fundamental step toward higher-order assemblies,^30^ questions remain regarding (i) the extent to which counterions stabilize these charged complexes, (ii) the influence of side-chain length on the initial packing behavior, and (iii) the contribution of solvent-mediated interactions to the overall thermodynamics of self-assembly.

Motivated by the work of Zhou et al.,^34^ we employed all-atom molecular dynamics (MD) simulations to systematically examine the dimer formation of four rigid amphiphilic molecules that share a common KTOF4 core but differ in side-chain length: KTOF4-C2, KTOF4-C6, KTOF4-C10, and KTOF4-C16 (Figure 1). Hereafter, these molecules are abbreviated as C2, C6, C10, and C16, respectively, with the numeric suffix indicating the number of carbon atoms per side chain. Each molecule contains eight side chains arranged as symmetric pairs attached at four distinct positions along the rod segment. Simulations were conducted in THF/water mixed solvents (15 and 33 vol % THF) containing tetrabutylammonium (TBA^+^) counterions to probe solvent effects and electrostatic stabilization. By analyzing the spatial distributions of solvent molecules and counterions around the self-assembled dimers, this study provides molecular-level insights into the solvent-mediated and counterion–driven interactions governing the self-assembly of rigid amphiphiles. Moreover, the influence of the side-chain length on the initial self-assembly stage is systematically investigated and correlated with the experimental observations, offering a deeper understanding of the structural factors underlying hierarchical assemblies’ formation.

**Figure 1:**
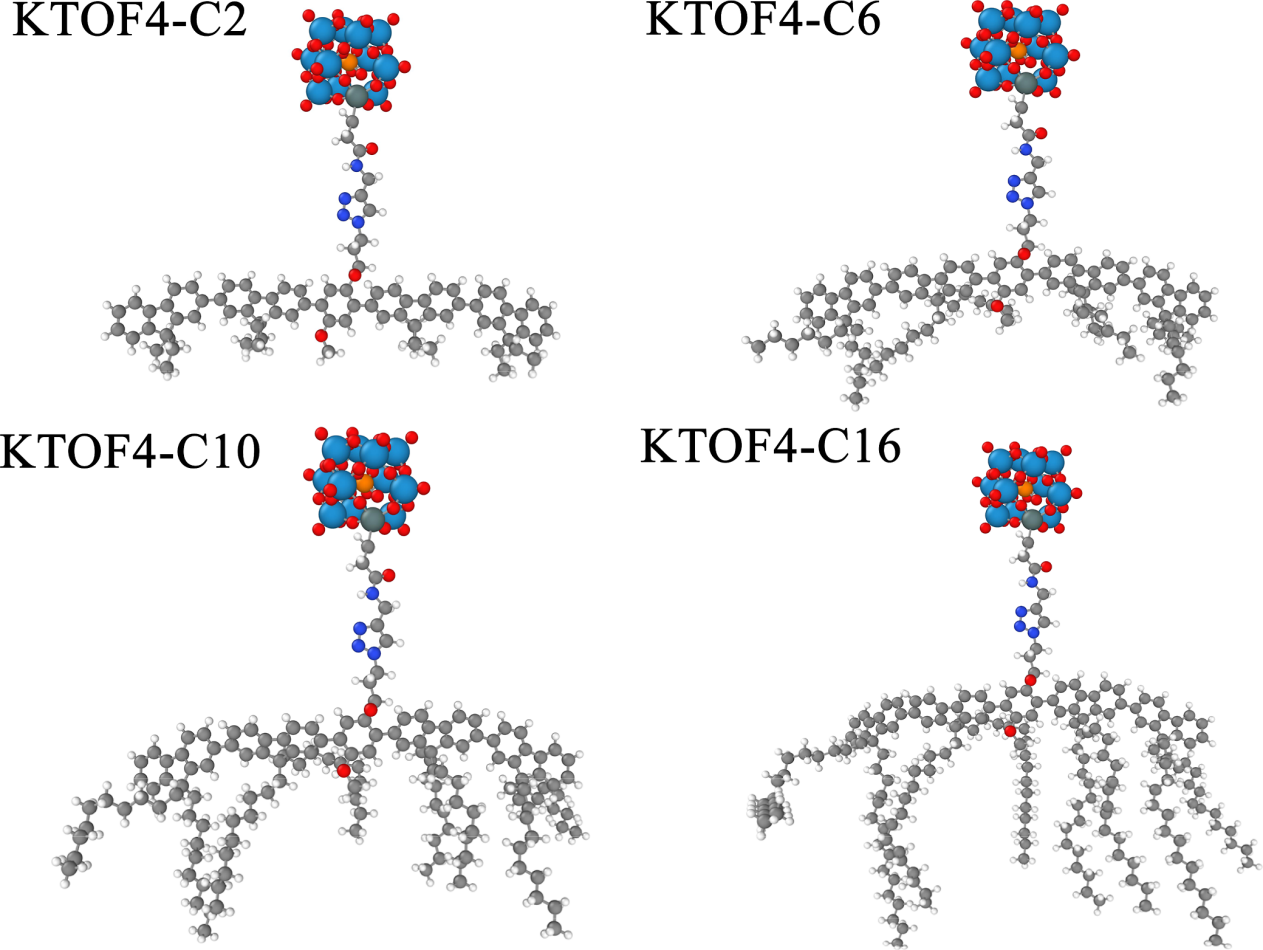
Molecular structure of rigid sphere-rod amphiphiles. The coloring of the atoms: Oxygen is red, hydrogen is white, nitrogen is dark blue, carbon is gray, tungsten is light blue, phosphorus is orange, and tin is dark gray.

## Materials and Methods

The structures of the Rigid Sphere-Rod Amphiphiles (RSRAs) studied here are shown in Figure 1. In our nomenclature, “K” denotes the Keggin polyoxometalate, “T” indicates the T-shaped nature of the molecules without “K”, “OF4” represents the 4 number of oligofluorene units linked together in the rod, and “C*” corresponds to the number of carbon atoms in each side chain attached to the rod.

The optimized structures of RSRAs, including the linker and rod components, the hydrophilic Keggin, tetrahydrofuran (THF), and tetrabutylammonium ions (TBA^+^), were generated using the Material Studio package.^35^ The OPLS-AA force field developed by Jorgensen et al. was employed to describe the inter- and intramolecular interactions for TOF4-C*, THF, and TBA^+,^ while the SPC/E model was selected for water.^36^ The SPC/E water model was selected because it provides a reliable description of both bulk water properties and interfacial hydration structure, which is important for accurately modeling water–macroion interactions at the charged Keggin surface in atomistic simulations. Due to limitations in the OPLS-AA force field for assigning charges to positively charged counterions, density functional theory was employed to accurately compute the partial charges for TBA^+^ (Table S1), while original OPLS-AA partial charges were assigned to TOF4-C* segments and THF. Following molecular optimization with the B3LYP/6-31G++(d,p) functional and basis set, CM5 charges were computed and assigned to the atoms of TBA^+^.^37,38^ Moreover, the force field parameters and partial charges for the Keggin POM were obtained from López et al.,^39^ which are compatible with OPLS-AA. The Keggin POM has an overall charge of −4.

Building on Zhou et al.,^34^ who observed self-assembly of KTOF4-C2 to KTOF4-C16 at THF fractions of 10–30 vol %, we probed dimerization at 15 vol % (within this range) and at 33 vol % (slightly above it). To ensure adequate sampling of self-assembled configurations, three independent simulations with different initial configurations were performed for all systems at 33 vol % THF. In addition, three independent simulations were carried out for the C2 system at 15 vol % THF. For the C6, C10, and C16 systems at 15 vol % THF, a single simulation was performed, as preliminary comparisons with the corresponding 33 vol % THF systems showed a qualitatively similar behavior. On this regard, additional independent replicas were not pursued for 15 vol % THF cases. In total, this resulted in more than 18 independent simulations. Each system consisted of 2 RSRAs, 8 TBA^+^ counterions, 4500 water molecules, and a variable number of THF molecules. The THF volume fraction (15 and 33 vol %) was adjusted by changing the number of THF molecules while keeping the number of water molecules fixed, with 177 THF molecules used for the 15 vol % systems and 500 THF molecules for the 33 vol % systems.

These components were randomly distributed within a cubic simulation box of initial dimensions 10.0 × 10.0 × 10.0 nm^3^ using the POLYMATIC package.^40^ All-atom MD simulations were performed using the LAMMPS software package.^41^ Periodic boundary conditions were applied in all directions to accurately model the bulk self-assembly behavior. An initial energy minimization was performed using the conjugate gradient method to remove unfavorable contacts. Each system was then equilibrated under isothermal–isobaric (*NPT*) conditions at 298 K and 1 atm for 5 ns where the density of the system reached the equilibrium value. The Lennard-Jones (LJ) interactions were truncated at a cutoff distance of 1.0 nm. The long-range Coulombic interactions were calculated using the particle–particle particle-mesh (PPPM) algorithm^42^ with an accuracy of 10^–4^, while the corresponding short-range cutoff distance was set to 1.0 nm.

Due to the rigidity of Keggin, the temperature of the Keggin atoms was controlled using a Nosé–Hoover thermostat within the rigid/*nvt* framework, while the Nosé–Hoover thermostat and barostat were applied to the rest of the system, employing damping parameters of 100 fs for temperature and 1000 fs for pressure control. The equations of motion were integrated by using the velocity-Verlet algorithm with a time step of 1 fs. The SHAKE algorithm^43^ was applied to constrain bonds and angles of water molecules, ensuring stable integration. The NPT equilibration resulted in simulation box size of around 5.9 × 5.9 × 5.9 nm^3^ for 33 vol % THF (5.5 × 5.5 × 5.5 nm^3^ for 15 vol % THF), and the density stabilized around 1.01 g/cm^3^ for 33 vol % THF and 1.03 g/cm^3^ for 15 vol % THF.

Production runs were performed in the canonical (*NVT*) ensemble at 298 K for at least 100 ns, with the final 50 ns of the trajectory being used for analysis to ensure reliable statistical sampling. Trajectory data were collected every 50 ps to analyze the structural properties and the dynamics of the amphiphilic assemblies, while thermodynamic properties were recorded every 1 ps to monitor the stability of the simulations. Visualizations were generated using visual molecular dynamics^44^ and OVITO.^45^

## Results and Discussions

### Interaction Energies

The driving forces underlying dimer formation among different RSRAs were elucidated by computing the total interaction energy between the two RSRAs after assembly. This total energy is a summation of electrostatic and van der Waals (vdW) interactions between all atomic pairs across the two RSRAs and averaged over the equilibrated dimer configuration in each simulation. No qualitative differences in self-assembly behavior were observed between the 15% and 33% cases, aside from the expected variations in the absolute energy values due to compositional differences. Thus, the discussion here focuses on the 33% case for clarity, although it could have equally been presented for the 15% case; the corresponding results for 15% are provided in the Supporting Information for reference. As noted earlier, three independent simulations were performed for each case, and the computed interaction energies for the 33% cases are summarized in Table 1. The three simulations for each case are ranked according to the total interaction energy between the RSRAs, from highest to lowest (C*-1, C*-2, and C*-3, respectively), and are consistently represented in all figures and graphs by blue, light orange, and green color, respectively.

**Table 1:**
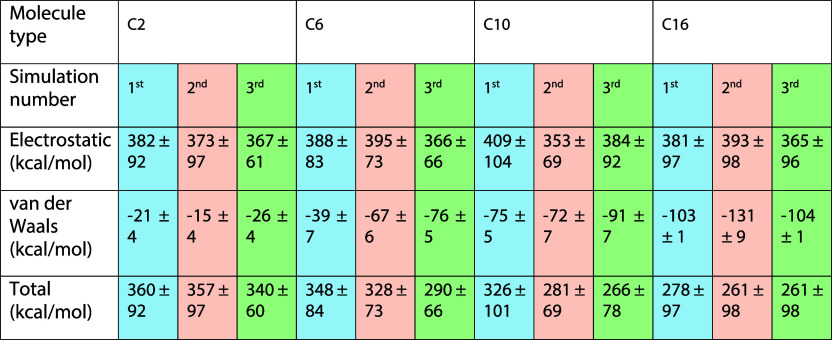
Time-Averaged Interaction Energies, Including Electrostatic, vdW, and Total Interaction Energies between Two RSRAs for the Four Different Cases in 33 Vol % THF^a^

a In these calculations, only two RSRAs are considered, and the solvents and counterions are excluded. For each case, we did three different independent simulations. These values were computed by averaging after dimer formation of the production runs.

The total interaction energies between the two RSRAs are positive across all cases, indicating that dimer formation is energetically unfavorable based on these components alone, as also shown in Table S2 for the 15% case. However, a clear trend emerges with increasing side-chain length as the total interaction energy becomes less positive, reflecting a shift toward more favorable interactions. While the electrostatic contributions remain strongly repulsive, the increasingly negative vdW interactions partially compensate for this repulsion. However, the magnitude of the vdW term is substantially lower than the electrostatic component, meaning that despite the enhanced hydrophobic interactions with longer side chains, electrostatic repulsion continues to be the dominant energetic factor.

Interestingly, when the Keggin units are excluded from the interaction energy calculations between the RSRAs, a noteworthy trend emerges, as summarized in Table 2. The total energy results indicate that increasing the length of the side chains generally makes dimerization more favorable when Keggin contributions are excluded. Energy decomposition shows that the electrostatic components are positive across all cases and therefore do not stabilize the dimer formation under these conditions. By contrast, the vdW contributions emerge as the favorable attraction between the RSRAs toward dimer formation. As shown in Figure 2, dimerization is accompanied by a decrease in inter-rod distance and a concomitant drop in vdW interaction energies, indicating that favorable vdW attractions between the hydrophobic blocks play an important role in stabilizing the dimer. The relatively short side chains in C2 offer limited favorable vdW interactions, making the approach to a stable dimer less straightforward, whereas longer side chains, as in C10 and C16, can transiently interdigitate before relaxing into the most stable configuration, illustrating how side-chain length influences the dimerization pathway. The results for 15 vol % THF are shown in Figure S1.

**Table 2:**
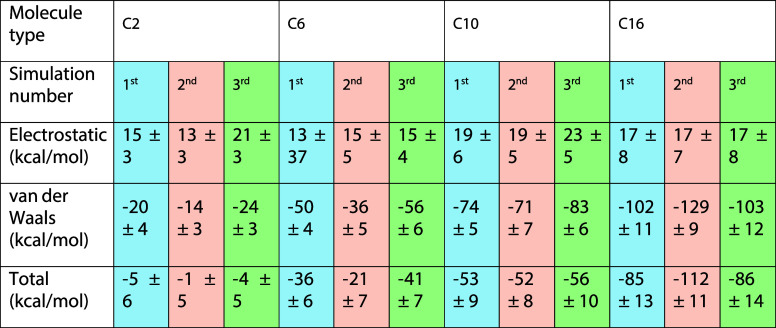
Electrostatic, vdW, and Total Energy between the Dimers without Including the Keggins, TBA+, and Solvents at 33 Vol % THF

**Figure 2:**
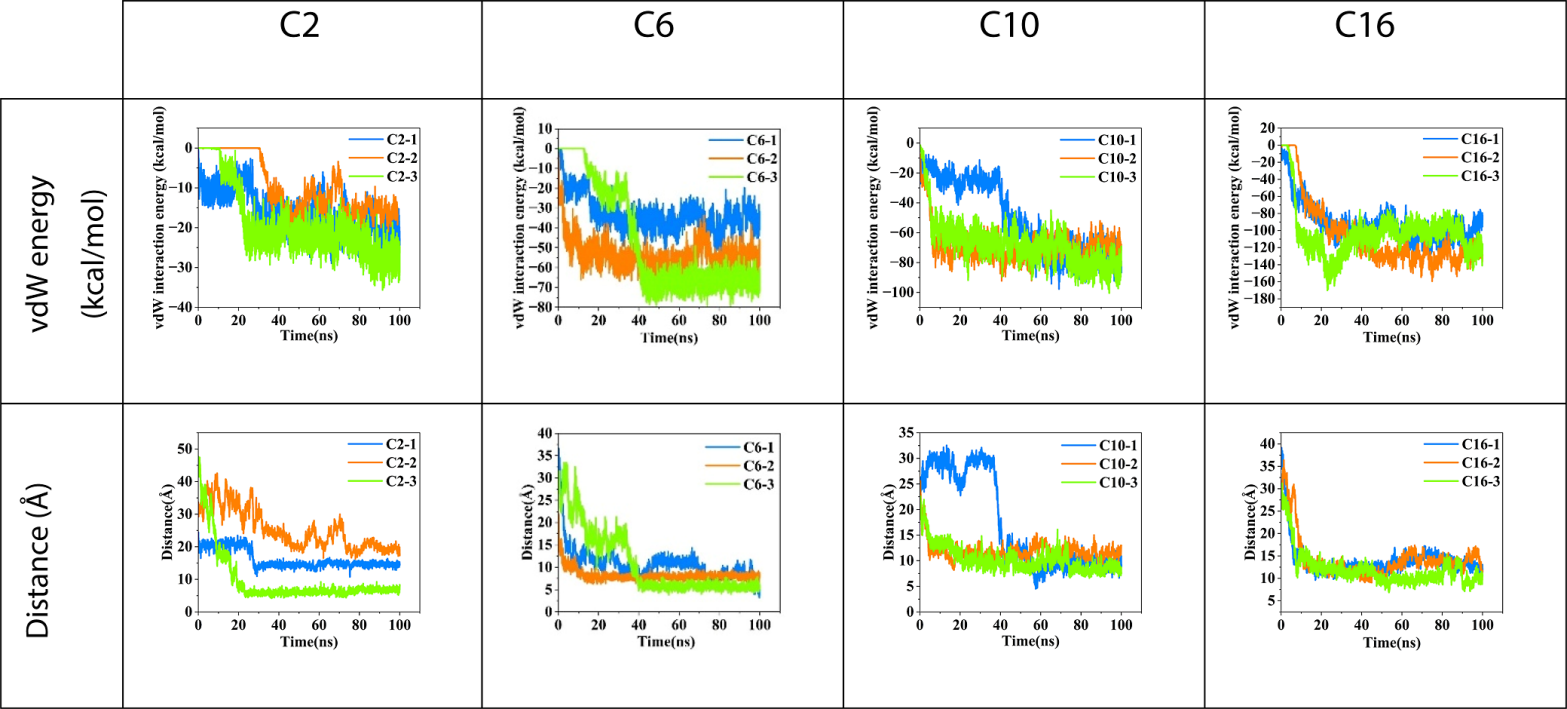
Intermolecular vdW interaction energies as a function of time between the RSRAs without including the Keggins, and center-to-center distance of rods as a function of time. For each case, the energy, and distances for three simulations in 33 vol % THF of that case are reported.

### Role of Hydrophobic Segments on the Dimer Structure and Packing Behavior

The packing behavior of the dimers was analyzed by computing geometric parameters that describe the relative arrangement of the two molecules during self-assembly. We focused on both the rod segments and their attached side chains as these define the dominant intermolecular vdW interactions. Specifically, we measured the horizontal and vertical separations between the central aromatic rings of the rod segments (Figure S2), as well as characteristic distances involving the side chains. In addition, we computed the relative orientations of two rods by calculating the angle between their principal axes. Each principal axis was defined as the line connecting the terminal carbon atoms at the ends of a rod segment (Figure S3), and the acute angle between the two axes (0–90°) was used to characterize their spatial alignment.

Our analyses indicate that the molecules preferentially arrange to maximize rod–rod and side-chain/side-chain interactions by decreasing both the horizontal and vertical separations between them, as illustrated in Figure 3 (Figure S5 for 15 vol % THF). The dimer structures with the lowest vdW energy (Figure 2) consistently are those with the shortest horizontal and vertical separations, indicating the stabilizing effect of close packing. Moreover, as summarized in Table 3, the side chains remain predominantly largely extended, as evidenced by their large average end-to-end distances, and they are also spread out, as reflected by the substantial distances between the ends of symmetric side chains, both before and after self-assembly (Table 4).

**Figure 3:**
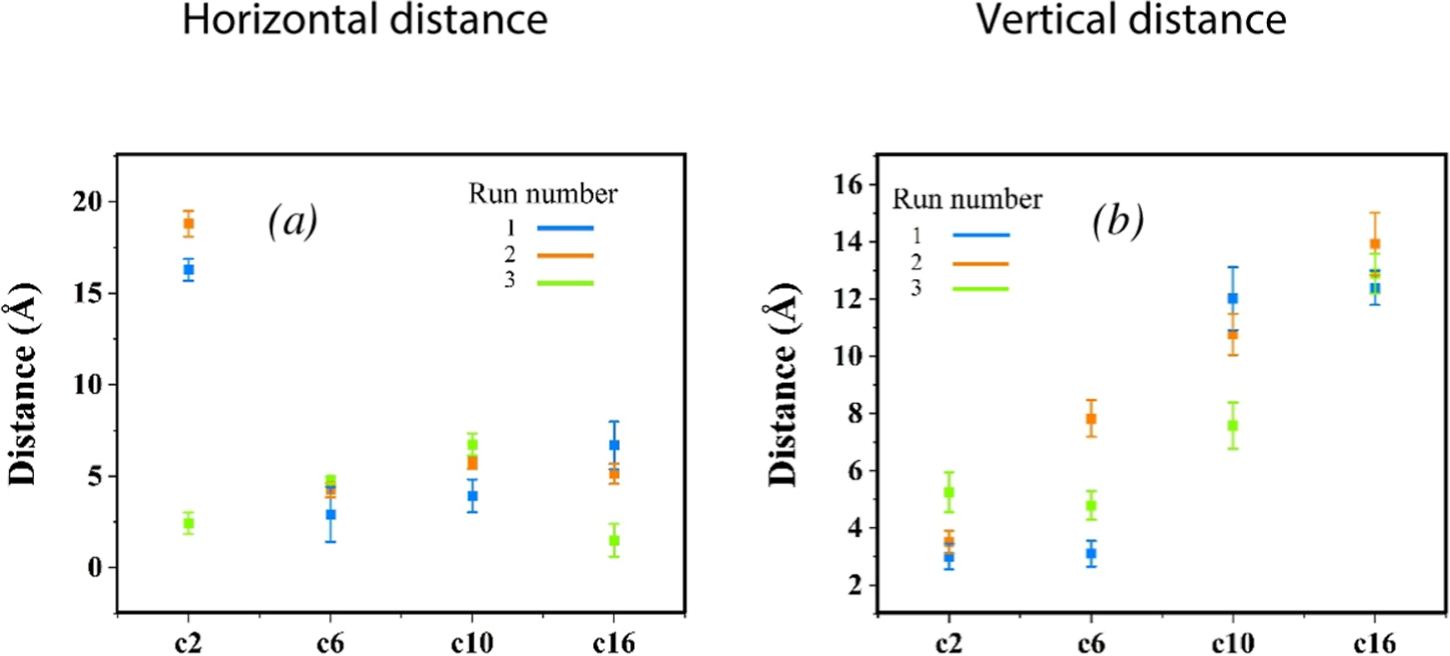
(a) Horizontal and (b) vertical distances between the central aromatic ring of the rods for 12 separate simulations at 33 vol % THF. Distances from independent runs are shown separately rather than averaged, as some trajectories populate distinct long-lived metastable configurations; averaging would obscure this multimodal behavior.

**Table 3:** Fully Stretched Length of the Side Chains and Average End-To-End Distance for Individual Side Chains for the Simulation with Lowest Total Energy in Each Case of 33 Vol % THF

		length of side chains after forming dimer (Å)	
	length of the fully stretched side chain (Å)	molecule 1	molecule 2
C2	∼1.54	1.54 ± 0.03	1.54 ± 0.03
C6	∼7.70	5.80 ± 0.50	5.82 ± 0.50
C10	∼13.86	9.74 ± 1.03	9.58 ± 1.05
C16	∼23.10	15.61 ± 1.66	14.43 ± 1.99

**Table 4:** End-To-End Distance of Collection of the Side Chains in C6, C10, and C16 Molecules^a^

		C6				C10				C16			
		side-chain collection number				side-chain collection number				side-chain collection number			
		1	2	3	4	1	2	3	4	1	2	3	4
end-to-end distance between the side chains	before self-assembly	13.66 ± 1.28	11.61 ± 0.82	12.61 ± 0.96	11.69 ± 1.15	18.83 ± 0.69	10.57 ± 1.57	11.74 ± 7.72	9.74 ± 2.39	11.75 ± 1.98	14.25 ± 2.52	7.91 ± 2.27	19.01 ± 1.42
	after self-assembly	12.94 ± 1.41	13.51 ± 1.04	8.79 ± 2.43	11.01 ± 2.57	16.70 ± 2.44	14.35 ± 3.26	14.49 ± 1.79	18.47 ± 3.03	20.57 ± 1.61	15.56 ± 3.12	13.14 ± 1.64	22.79 ± 2.84

a The distance is calculated between the last atoms of each side chain in the collection. Collections are numbered sequentially from 1 to 4, with 1 and 4 located at the terminal ends of the rods and 2 and 3 positioned in between. The schematic of side-chain collections and their corresponding end-to-end distances is illustrated in Figure S4. As the lengths of the side chains in C2 molecules are too short and there is no significant difference between the side chains in each collection, we did not mention their value in this table.

A closer examination of the packing structures of dimers with relatively higher vdW energies (representative final configurations are shown in Figure 4) reveals that these correspond to cases where the two molecules deviate substantially from a parallel orientation. Such deviations arise either from constraints imposed by the linker containing the Keggin unit, as in the C2-1 case, or from the failure of the side chains to interdigitate, leading instead to side-by-side packing, as in C16-2. These observations suggest that deviations from parallel packing compromise efficient rod–rod interactions and reduce the overall stability of the dimer. Moreover, structural deviations were systematically quantified by calculating the angle between the rod segments, with the results shown in Figures 5 and S6 for 33 and 15 vol % THF, respectively.

**Figure 4:**
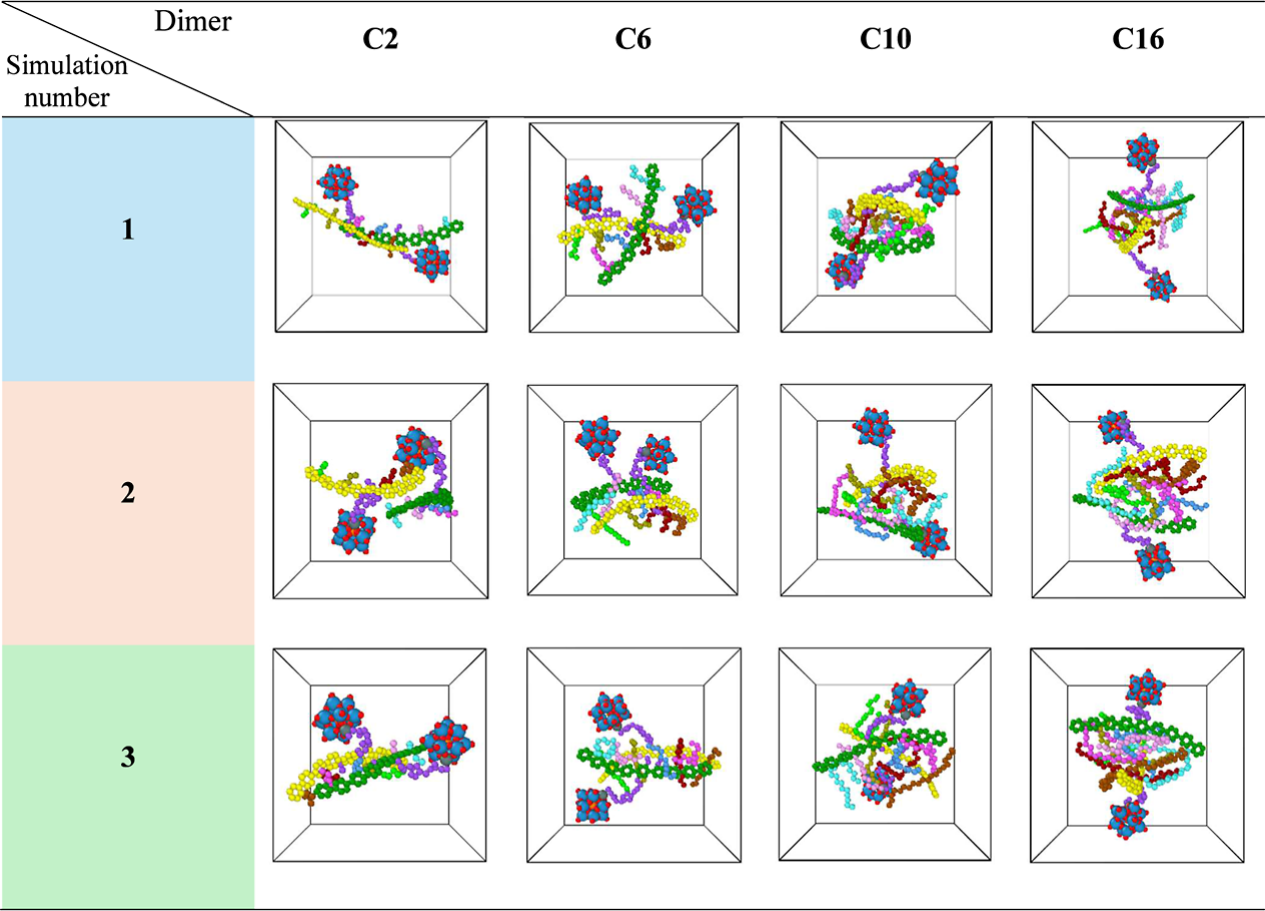
Final configuration of dimer in 12 different simulations at 33 vol % THF. For clarity, the rods are colored in green and yellow, the linker in purple, the tungsten of Keggin in blue, oxygens of the Keggin in red, phosphorus in orange, and the side chains in different colors (cyan, pink, blue, and dark pink for one molecule, and green, beige, dark red, and brown for other molecules). The solvent and counterion molecules are not visualized.

**Figure 5:**
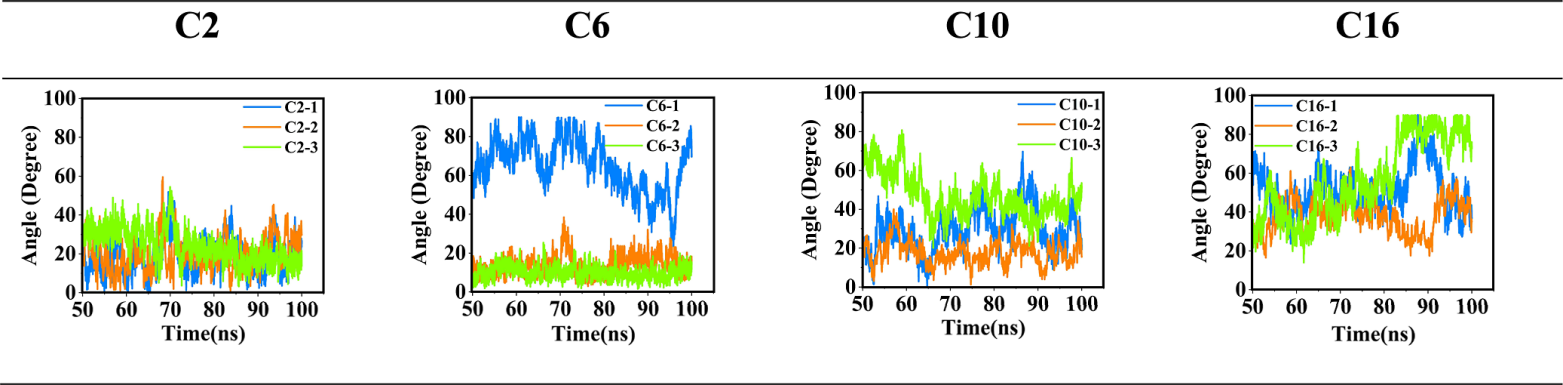
Angle between the rods after forming the dimers for simulations at 33 vol % THF.

Angular analysis enables an in-depth examination of the relationship between rod orientation and vdW stability. For the C2 and C6 systems, nearly perfect parallel alignment coincides with the lowest-energy dimer structures, emphasizing the importance of close rod–rod contact at short side-chain lengths. By contrast, for systems with longer side chains (C10 and C16), strict parallel orientation is not always required to achieve low-energy states provided that the rods retain a stacked arrangement with close horizontal proximity between the central aromatic rings. For instance, in the C16 system, a pronounced deviation from parallel orientation is observed (Figure 5), accompanied by a vertical separation between the central aromatic rings that is significantly smaller than the combined end-to-end lengths of the opposing side chains, indicating extensive interdigitation. This interdigitation becomes a key stabilizing factor in longer side-chain systems and signals a transition toward more disordered packing within the layer. Further analysis of side-chain extension for longer side-chains shows that those attached at the terminal sites of the rods (collections 1 and 4) are more stretched and remain less interdigitated, while those at the inner sites (collections 2 and 3) penetrate more deeply into the opposing molecule and exhibit pronounced interdigitation (Table 4). This spatial variation emphasizes the asymmetric role of side chains in stabilizing dimer packing: terminal chains primarily extend outward, whereas inner chains interlock to reinforce cohesion within the dimer.

### Solvent Organization around Hydrophobic Rods

An important aspect of dimerization is how solvent molecules reorganize in response to the close packing of amphiphilic components.^31^ To evaluate this effect, we computed the spatial distribution function (SDF) of solvent molecules around the rod segments. As shown in Figure 6a for the case of C6-3, the region between the two rods is essentially devoid of solvent, with only a low density of THF molecules being observed. This pronounced depletion indicates that solvent molecules are expelled from the space between the two rods, thereby promoting stronger hydrophobic interactions between the rods.

**Figure 6:**
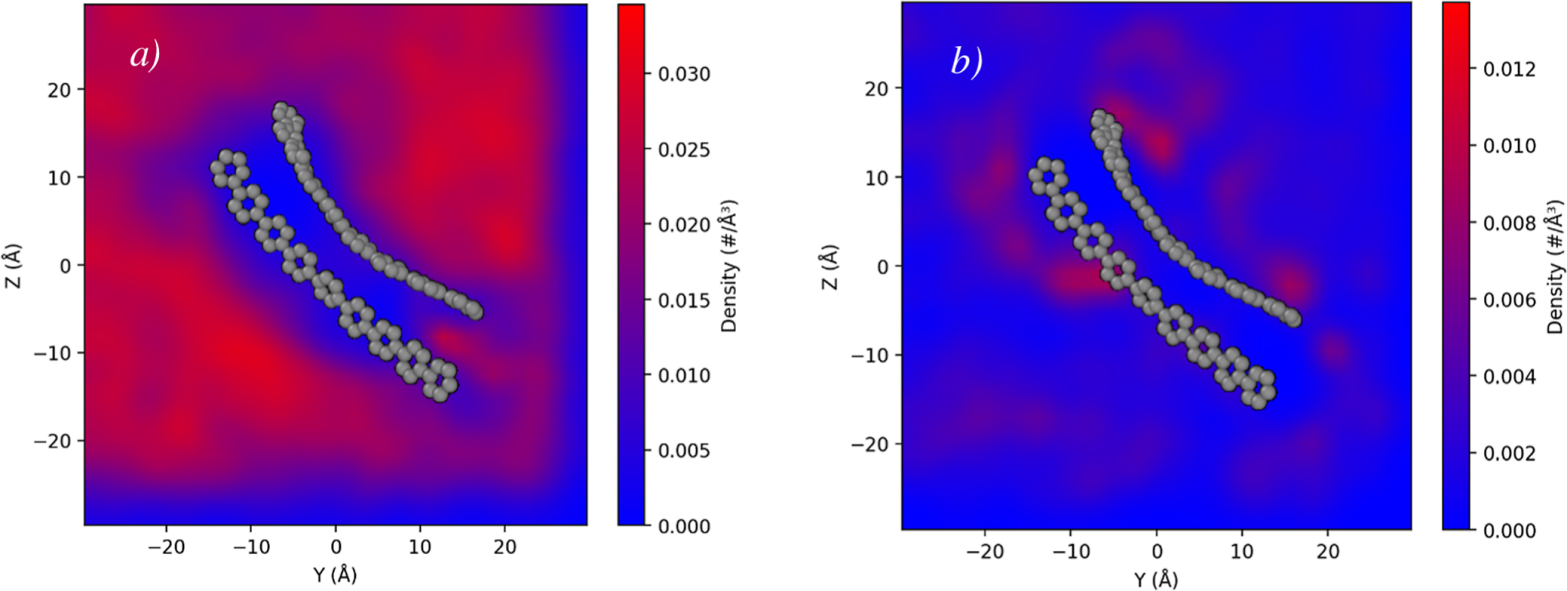
SDF of (a) water molecules and (b) THF molecules around the rods in C6-3 dimer at 33 vol % THF. For clarity, only the rods of the molecules are visualized on the heatmap of SDF.

As shown in Figure S7, the interaction energy between water and rod + side-chain segments is unfavorable, and it is because of the hydrophobic nature of those segments. As a result, water is displaced from the space between them, making the hydrophobic–hydrophobic interaction energies substantially more favorable after dimerization.

Furthermore, the SDF analysis shows that THF molecules accumulate preferentially along the outer surfaces of the rods, forming a stabilizing solvation shell (Figure 6b). This observation is consistent with the favorable interaction energies between THF and the hydrophobic segments, which become increasingly negative as side-chain length increases (Figure S8), with average values of −149 ± 44, −189 ± 53, −217 ± 61, and −261 ± 71 kcal/mol for C2, C6, C10, and C16, respectively. Although the concentration of THF molecules in the simulations is relatively low, their role in solvating the rod exteriors and supporting dimer stability is considerable. Similar solvation shell formation is evident for C2, C10, and C16 dimers, as shown in Figure S9. As the length of the side-chains increases, a larger portion of the space between the rods becomes occupied by the side-chains themselves, resulting in a greater solvent-excluded region.

Together, these results reveal a cooperative solvent reorganization mechanism: water is displaced from the rod–rod interface as hydrophobic contacts strengthen, while THF molecules preferentially organize along the outer surfaces of the amphiphiles, forming a solvation shell that further stabilizes the dimer structure.

### Counterion Mediation

Revisiting Tables 1 and S2 reminds us that although hydrophobic interactions contribute favorably to dimer stability, they are considerably weaker than the strong electrostatic repulsion between the negatively charged Keggin units. Across the four dimer systems, the electrostatic interaction energies are consistently large and repulsive, averaging 380 ± 73 kcal/mol. A closer inspection reveals that the dominant contribution arises from direct Keggin–Keggin interactions, which alone average +543 ± 83 kcal/mol. This large repulsive force, stemming from the negative charges of the Keggins, would make stable dimer formation highly unfavorable in the absence of an additional mediating mechanism.

The persistence of the stable dimers under such conditions reveals the decisive role of the TBA^+^ counterions. For a detailed understanding, the total interaction energies between the dimers and the TBA^+^ counterions were computed for all simulations, with the results shown in Figures 7 and S10 for 33% and 15%, respectively. In every case, TBA^+^ counterions provide strong attractive interactions with negatively charged Keggins, effectively screening the electrostatic repulsion and converting the overall dimer interaction energies into favorable values. This conclusion is further supported by Keggin-TBA^+^ distance analysis, which quantifies the characteristic distances between counterions and the Keggin units.

**Figure 7:**
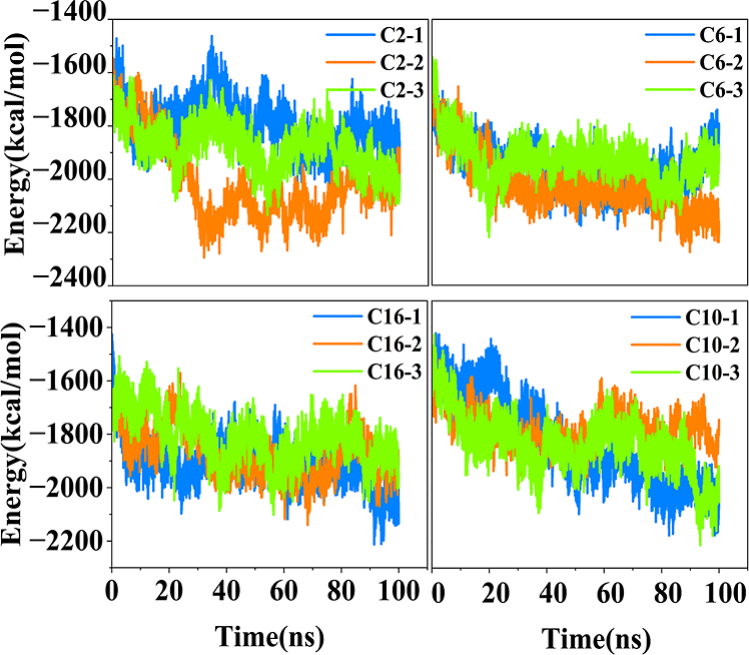
Total interaction energy between the dimers and TBA^+^ counterions for all 12 simulations in 33 vol % THF.

This observation is consistent with prior experimental and computational studies of highly charged systems,^30,46–49^ which demonstrate that counterion mediation is essential in reducing electrostatic repulsion and enabling self-assembly. Further analysis of the interaction energies (Table S3) confirms that the dominant attractive contribution originates from TBA^+^-Keggin interactions, which are comparable across all systems.

### Counterion Organization around Hydrophilic Keggins

The spatial arrangement of the TBA^+^ counterions was characterized by calculating the time-averaged distance between the nitrogen atom of TBA^+^ and the central phosphorus atom of Keggin following dimer formation. This metric provides a direct measure of the characteristic Keggin–TBA^+^ separation and is particularly suitable for the present systems, which contain a limited number of counterions, for which conventional radial distribution functions (RDFs) can be sensitive to normalization and correlation effects. As summarized in Table S4, the TBA^+^ ions predominantly reside at an average distance of 8.5 ± 0.4 Å from the Keggin center. The narrow error bars indicate that counterions consistently maintain this preferred position, creating a stable screening shell around negatively charged Keggins. By maintaining this average distance, the counterions effectively screen the otherwise prohibitive Keggin–Keggin repulsion and thereby play a pivotal role in stabilizing the dimer state.

### Solvent Organization around the Keggins

RDFs of water and THF molecules relative to the center of Keggin were computed to characterize the solvation environment around negatively charged Keggins. The schematic representation of the distance definition is depicted in Figure 8a. Representative results from the C6-3 simulation (Figure 8b) demonstrate solvation features that are consistent across all systems.

**Figure 8:**
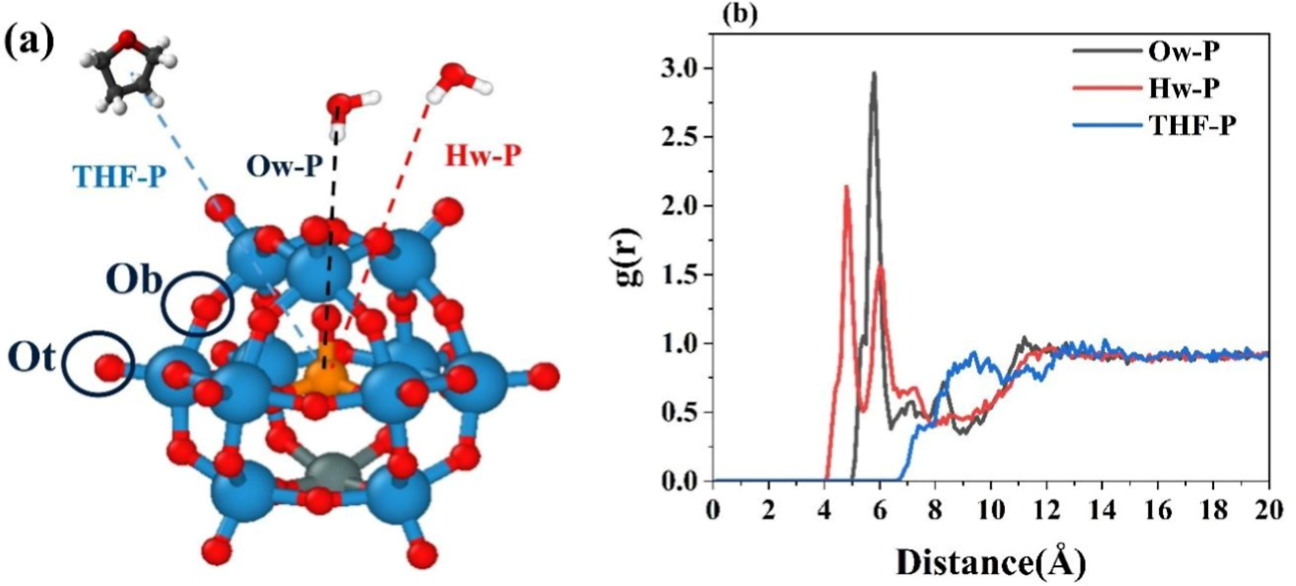
(a) The schematic of bridge oxygen and terminal oxygen and distances between the solvent molecules and the center of Keggin. (b) The RDF of water oxygen (Ow), water hydrogen (Hw), and the center of mass of THF around the center of Keggin (P).

For the hydrophilic Keggins, the first solvation shell is dominated by water molecules. The RDF peak in Figure 8b indicates that water hydrogen atoms are located at an average distance of ∼4.5 Å from the Keggin center. Considering the Keggin radius of ∼5 Å (measured across terminal oxygens), this implies that water molecules position themselves sufficiently close to interact not only with terminal oxygens but also with bridge oxygens (Ob). The relatively higher negative charge on the bridging oxygens enhances their ability to form hydrogen bonds with water molecules, contributing to the formation of a tightly bound hydration shell. Hydrogen bonding analysis (Table 5) confirms this interpretation: although steric effects limit access to bridge oxygen sites, both terminal and bridge oxygens participate in hydrogen bonding. Approximately 14 hydrogen bonds are formed by terminal oxygens, whereas bridge oxygens contribute about 9 hydrogen bonds per Keggin, yielding an average of ∼23 water-Keggin hydrogen bonds.

**Table 5:** Average Number of Hydrogen Bonds between Water Molecules and Ot and Ob of Keggins for the Systems with Lowest Total Energy (Table S5)­^a^

RSRA	avg. no. of H-bond	avg. no. of H-bond
	per Keggin (Ot)	per Keggin (Ob)
C2-3	14.52 ± 0.17	8.34 ± 0.33
C6-2	13.33 ± 0.67	8.40 ± 0.37
C10-1	14.78 ± 0.72	9.32 ± 0.40
C16-2	15.84 ± 0.61	9.56 ± 0.39

a Each Keggin has 12 Ot and 24 Ob.

The solvation behavior of THF around Keggins differs markedly from that of water. As shown in Figure 8b, the RDF shows no sharp peaks but rather a broad distribution, consistent with weaker, less structured interactions. On average, THF molecules form a secondary solvation shell at ∼9 Å from the Keggin center, beyond the tightly bound hydration layer. This hierarchical solvation pattern reflects the strong electrostatic attraction between water and the negatively charged Keggin (reinforced by hydrogen bonding), the screening effect of bulky TBA^+^ counterions (Figure 7), and the weaker polarity of THF (dielectric constant ∼ 7). As a result, THF molecules predominantly populate the outer regions, forming a diffuse, bulk-like solvation environment rather than a well-defined hydration shell.

To probe solvation dynamics, we computed the residence time of water molecules in the first solvation shell (Ow-P distance ≤6.5 Å) using a 12 ns trajectory with 10 fs frequent damping for the C6-3 system. The autocorrelation function (ACF) of water residence time around the Keggin was computed and is provided in Figure S11. The ACF was fitted with the Kohlrausch–William–Watts (KWW) stretched-exponential function C(tτ)=A0exp[−(tτ)β] , where τ is a characteristic relaxation time and β is a measure of the deviation from exponential behavior. The mean residence time ⟨τ⟩ is given by $\left\langle \tau \right\rangle =\int_{0}^{\infty }C\left(t\right)dt=\frac{\tau }{\beta }\Gamma \left(\frac{1}{\beta }\right)$ where Γ­(*x*) is the gamma function. The average residence time is approximately 648 ps. This extended residence is significantly longer than the few picoseconds typically observed for bulk water,^50–52^ and it is similar to water residence time in the vicinity of charged surfaces, which is in the order of hundreds of picoseconds.^53^ The long residence arises from the high charge density of the Keggins and the strong hydrogen bonding at both bridge (Ob) and terminal (Ot) oxygens, which together tend to freeze the motion of water molecules near the surface.

### Dynamics of RSRAs

Beyond solvent organization, we assessed how the RSRAs adapt to the surface during dimerization. Root mean squared deviation (RMSD) of individual amphiphiles during the final 50 ns of production runs corresponding to the postassembly, equilibrated regime shows an increasing trend with side-chain length: 5.13 ± 0.27 Å (C2), 6.41 ± 0.27 Å (C6), 8.28 ± 0.25 Å (C10), and 9.23 ± 0.20 Å (C16) (the RMSD during the all production time from 0 to 100 ns is shown in Figure S12). The larger deviations for longer side chains reflect their greater conformational flexibility and the capacity to reorient during packing. Additional deviations also arise from the dynamics of the flexible linker between the Keggin headgroup and the rod segment, which is adjusted dynamically in response to solvent and dimerization forces.

Finally, mean squared displacement (MSD) analysis of the dimers for the 33% case (Figure 9), calculated from the equilibrated portion of the trajectories, reveals that the side-chain length modestly influences relative dimer mobility. Dimers with longer side chains exhibit lower self-diffusion coefficients, consistent with their increased molecular size and reduced translational freedom. Because each system contains only a single dimer, long-time MSD data become increasingly noisy. Accordingly, while the MSD was evaluated over the final 15 ns of the equilibrated trajectories, the slopes used to characterize relative mobility were obtained from the initial 4 ns of the time-averaged MSD, where the linear regime is most robust and least affected by statistical noise. Systems with longer side chains exhibit lower self-diffusion coefficients, consistent with their increased molecular size and reduced translational freedom. Also, according to the results for 15 vol % THF (Figure S13), the same trend is observed, where increasing the side-chain length decreases the dynamics of the dimers. However, the self-diffusion coefficients in 15 vol % THF are nearly twice those in 33 vol % THF, indicating that dimers in water-rich mixtures (15 vol % THF) are more mobile due to the lower solvent quality for the hydrophobic segments, which drives the dimers to avoid the solvent by increasing their dynamics.

**Figure 9:**
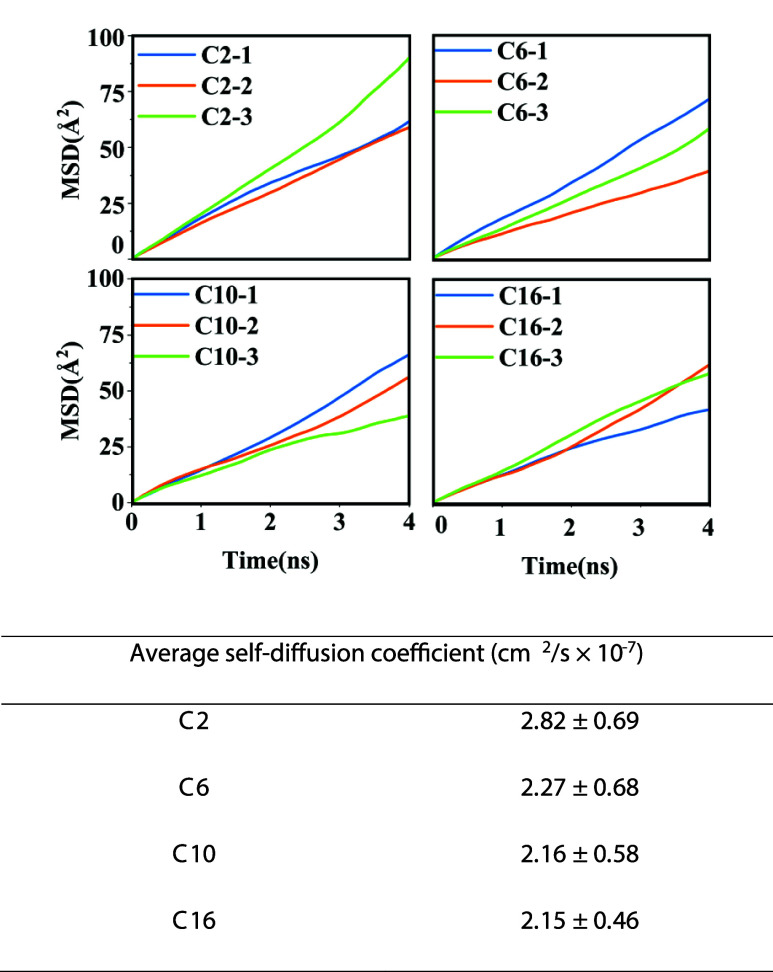
Comparison of MSD of the dimers and the average self-diffusion coefficient across the three runs for each case in 33 vol % THF. MSD analysis was performed using the final 15 ns of the production trajectories, during which the dimers remain structurally stable. Diffusion coefficients were estimated from the short-time linear regime (4 ns) of the time-averaged MSD, where statistical noise is minimal and the displacement sampling is most reliable for a single diffusing object.

## Conclusion

Dimerization of rigid sphere-rod amphiphiles (RSRAs) arises from a cooperative balance of forces: strong POM–POM electrostatic repulsion is offset by counterion screening from TBA^+^, while hydrophobic rod/side-chain contacts (enhanced by increasing side-chain length) supply the cohesive attraction that locks dimers in place. When Keggin contributions are omitted, the vdW term clearly drives association, explaining why longer side chains promote stabilization via a closer rod proximity and interdigitation. Packing correspondingly shifts from near-parallel rod alignment (short chains) to tilted, interdigitated arrangements (long chains) without sacrificing the stacked contact between central aromatic rings.

Solvent reorganization is integral to this stabilization. Water is depleted from the inter-rod region as hydrophobic contacts form, while THF accumulates along the exterior hydrophobic surfaces as a loose solvation shell. Around Keggin, a structured hydration layer persists. Both terminal and bridge oxygens participate in hydrogen bonding, and water exhibits long residence times. TBA^+^ counterions occupy preferred distances from the Keggin center, forming a screening shell that reduces the otherwise prohibitive POM–POM repulsion. These features are robust across 15 and 33 vol % THF, with expected shifts in magnitudes but unchanged qualitative trends.

Single-molecule RMSD increases with side-chain length, reflecting greater conformational freedom and linker motion, while dimer self-diffusion decreases modestly as the size and interdigitation grow. Together, these results provide an atomistic blueprint linking side-chain architecture, counterion organization, and solvent structure to the thermodynamic and dynamic stabilization of RSRA dimers (the initial step toward different self-assemblies observed experimentally and computationally).

Practically, the work suggests tunable levers for design: side-chain length to control packing geometry and vdW cohesion; solvent polarity to modulate interfacial water depletion and THF solvation; and counterions for tuning screening strength. Future studies will extend beyond dimerization to examine self-assembly involving larger numbers of amphiphilic molecules, capturing the cooperative pathways that lead from dimers to multilayered vesicles. Both all-atom simulations and coarse-grained models will be valuable for bridging molecular detail on longer time and length scales. In the future, using a chemistry-based coarse-grained model, we will explore different solvent environments and counterion chemistries and will reveal how polarity and ion identity modulate stabilization.

## Supplementary Material



## Notes

Additional figures, tables, and computational details (PDF)
