# Supplementary material for: Role of Side-Chain Length and Counterion Mediation on Dimerization of Rigid Sphere-Rod Amphiphiles: A Molecular Dynamics Investigation
Source: Langmuir. 2026 Jan 24;42(4):3468–78. doi: 10.1021/acs.langmuir.5c05734 (PMC12874539; doi:10.1021/acs.langmuir.5c05734)
Supplement: Supplementary file 1 [file la5c05734_si_001.pdf]

Supporting information

The Role of Side-Chain Length and Counterion Mediation on Dimerization of Rigid Sphere-Rod Amphiphiles: A Molecular Dynamics Investigation

Farzad Toiserkani[a], Yifan Zhou[a], Abdol Hadi Mokarizadeh[a], Javad Tamnanloo[a], Tianbo Liu[a], and Mesfin Tsige[a]*

[a] School of Polymer Science and Polymer Engineering, the University of Akron, Akron, Ohio 44325 United States. Email: mtsige@uakron.edu

Table S1. Atom types and corresponding calculated charges of TBA+ counterion. Only the charges on one chain and the N atom are represented here. There are four types of Carbon atoms (C1, C2, C3, and C4), and four types of Hydrogen atoms (3*H1, 2*H2, 2*H3, and 2*H4) on each chain. The total charge of the TBA+ counterion is +1.

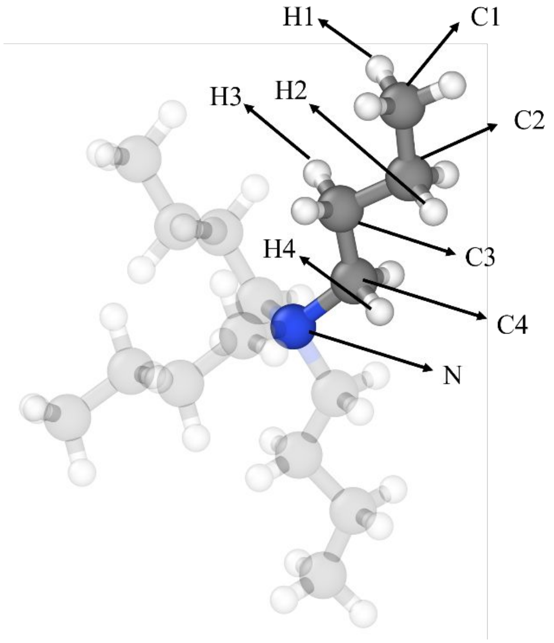

Atom Type	Calculated charge
C1	-0.24
C2	-0.15
C3	-0.14
C4	-0.04
H1	0.08
H2	0.075
H3	0.09
H4	0.15
N	-0.2

Table S2. Time averaged interaction energies, including electrostatic, van der Waals, and total interaction energies between two RSRAs for the four different cases in 15 vol% THF. C2 case has three different independent simulations and for other cases single simulation has been done. These values were computed by averaging after dimer formation of the production runs.

Molecule type	C2			C6	C10	C16
Simulation number	1st	2nd	3rd	1st	1st	1st
Electrostatic (kcal/mol)	540±90	440±96	468±88	428±78	485±101	433±96
vdW (kcal/mol)	-4±1	-39±6	-12±4	-49±7	-72±6	-155±19
Total (kcal/mol)	536±90	402±96	456±89	380±78	413±101	279±97

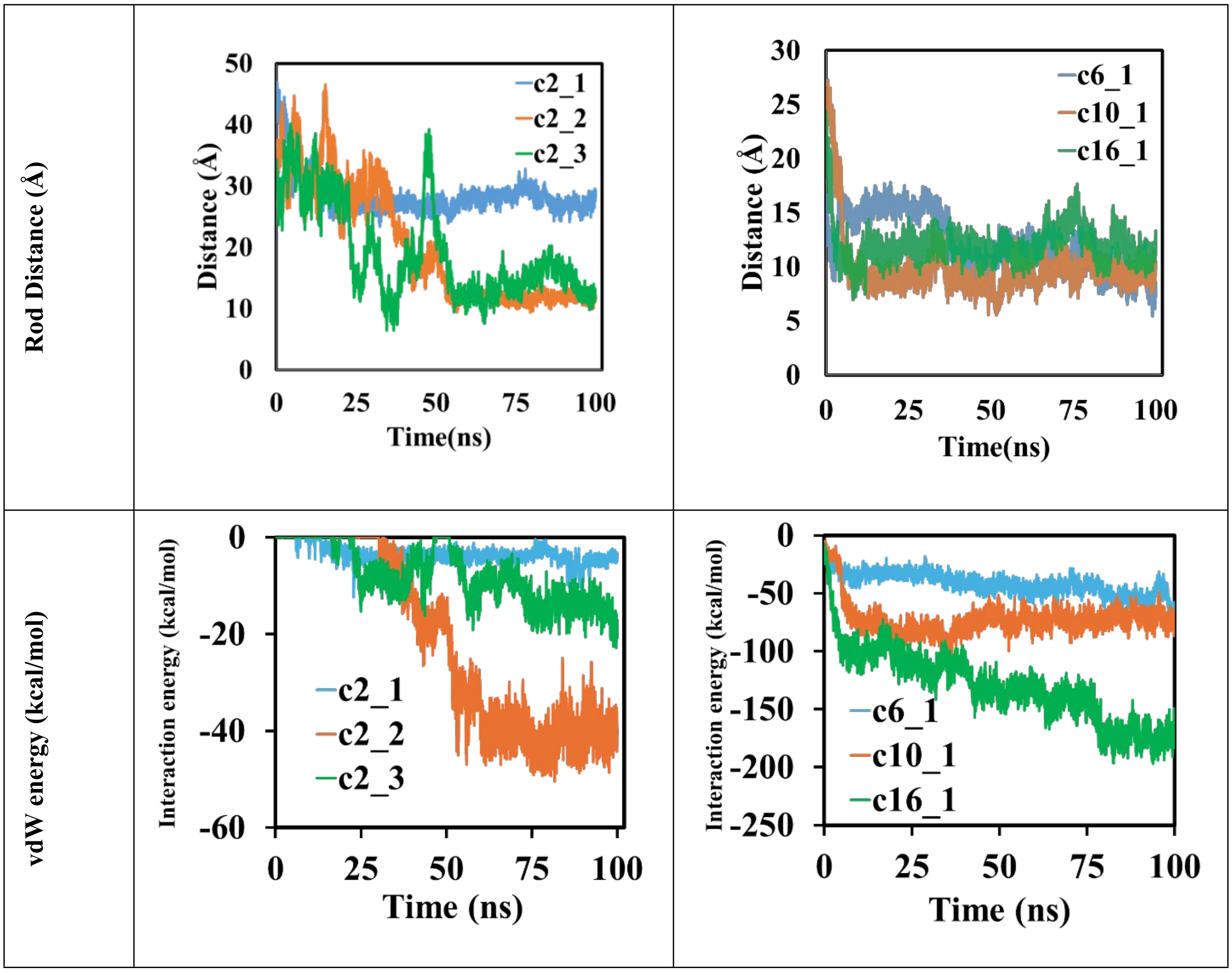

Figure S1. Intermolecular vdW interaction energies as a function of time between the RSRAs without including the Keggins, and Center-to-center distance of rods as a function of time for 15 vol% THF simulations. The C2 cases are reported separately as that case has three separate simulations.

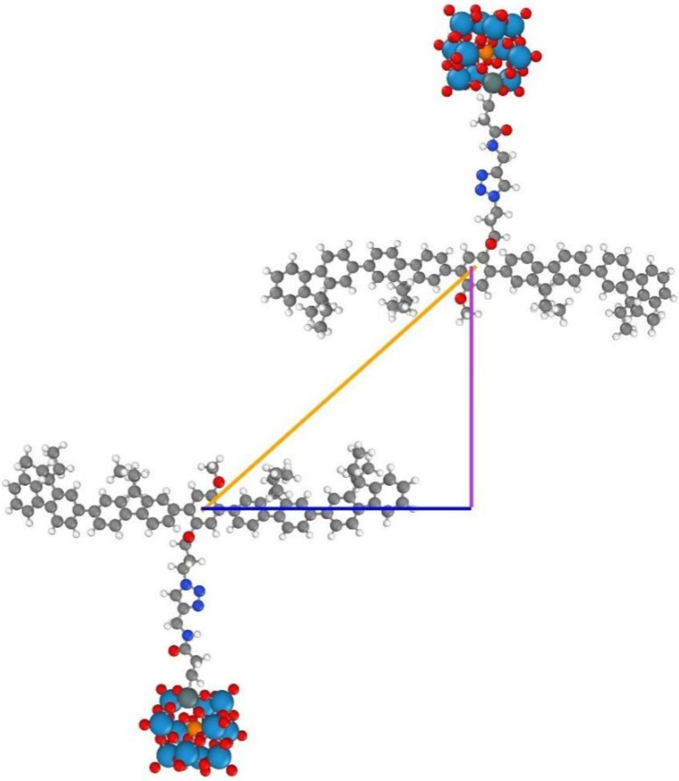

Figure S2. The rod center-to-center distance. The Yellow line is the actual distance, blue line is the horizontal component of the distance, and the purple line is the vertical component of the distance

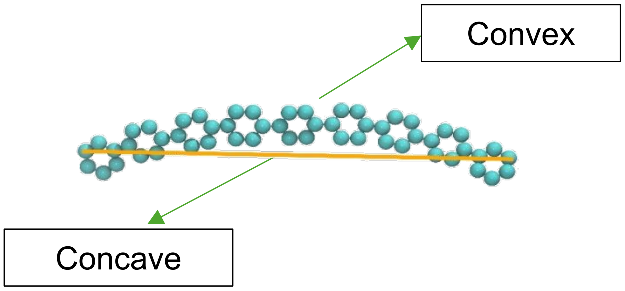

Figure S3.Defined line between both ends of the rod and schematic of convex and concave sides of them.

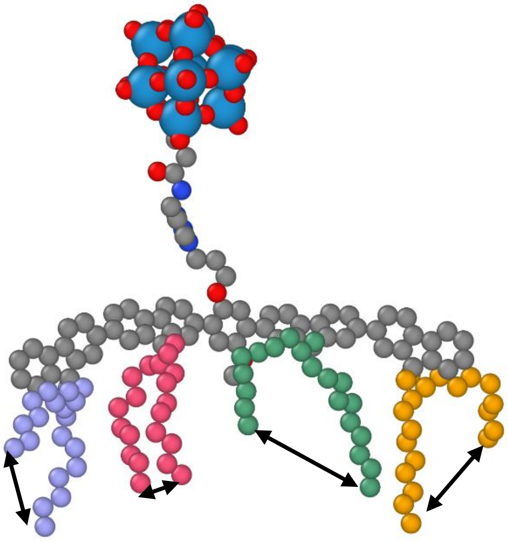

Figure S4. Illustration of side-chain collections and the definition of their corresponding end-to-end distances. Side-chain collections are numbered sequentially along the rod axis, starting from one end of the rod (purple side chains) and proceeding to the opposite end (orange side chains). Accordingly, collections 1, 2, 3, and 4 correspond to the purple, pink, green, and orange side chains, respectively. The end-to-end distance for each collection is defined as the distance between the terminal atoms of the side chains within that collection which are shown in black arrows.

Horizontal Distance

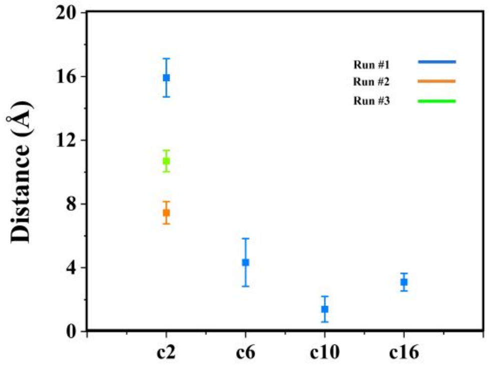

Vertical Distance

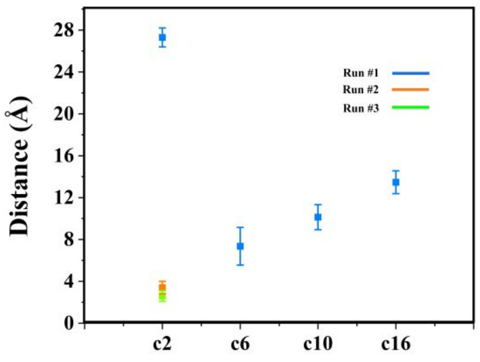

Figure S5. Horizontal and vertical distance comparison between C2 to C16 simulations at 15 vol% THF

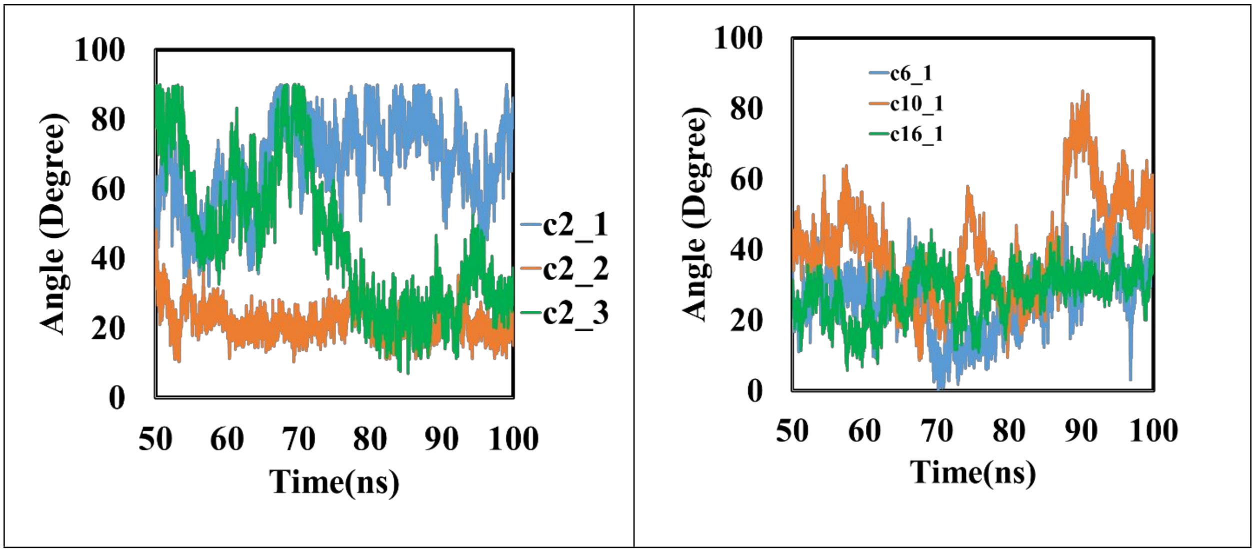

Figure S6. Angle between the rods as a function of time after forming the dimer for 15 vol% THF.

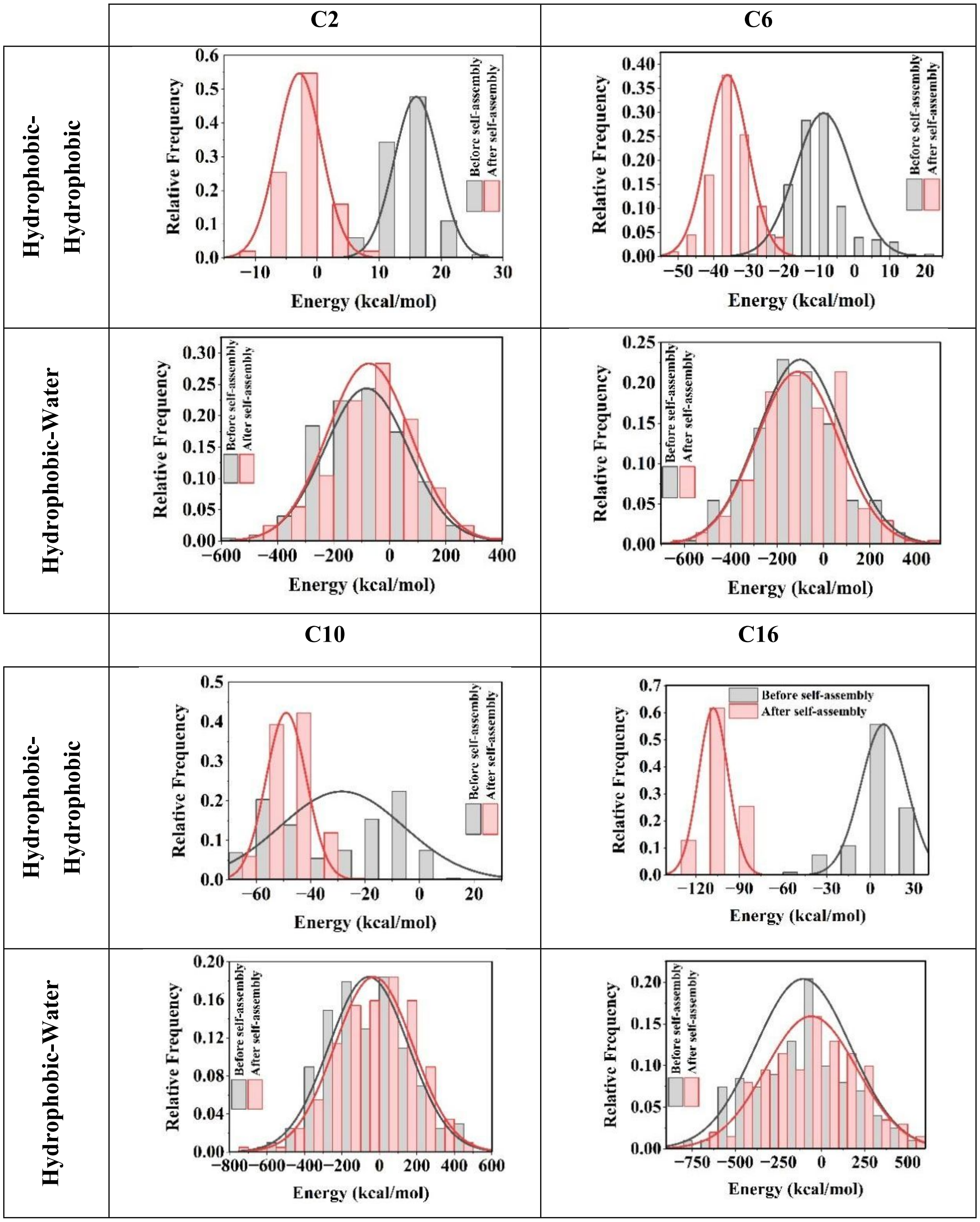

Figure S7. The total interaction energy between the hydrophobic-hydrophobic segments, and water molecules and hydrophobic segments of the RSRAs for the systems with lowest total energy

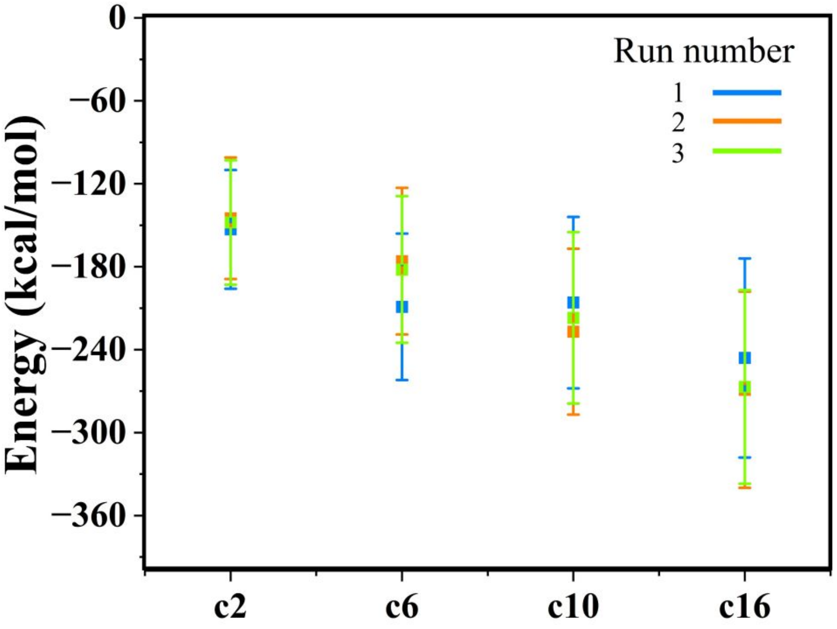

Figure S8. The total interaction energy between the THF and hydrophobic segments in 33 vol% THF.

	Water heatmap	THF heatmap
**C2-3**	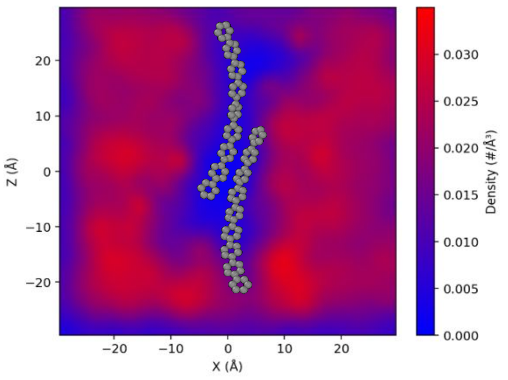	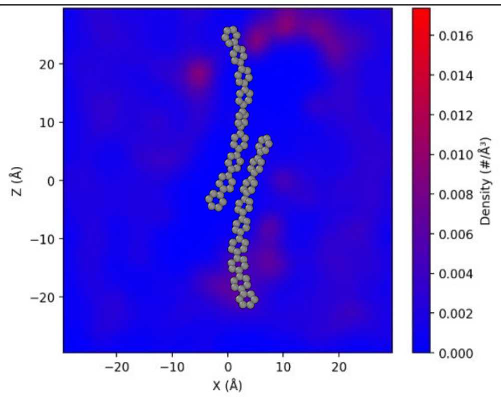
**C10-3**	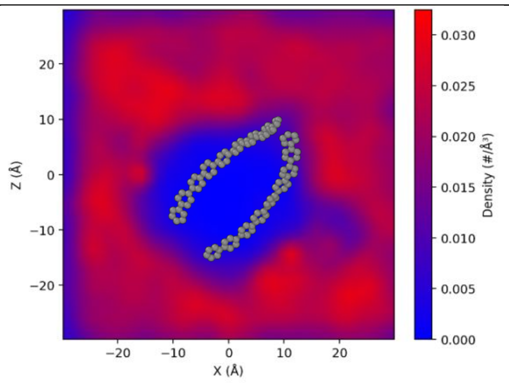	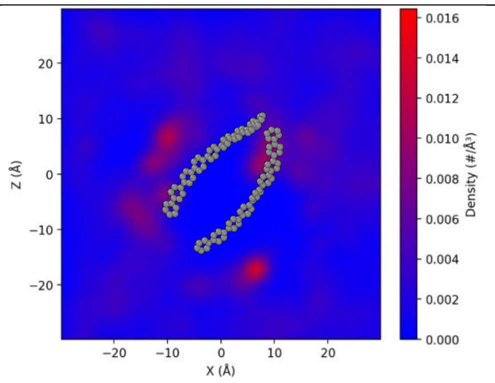
**C16-3**	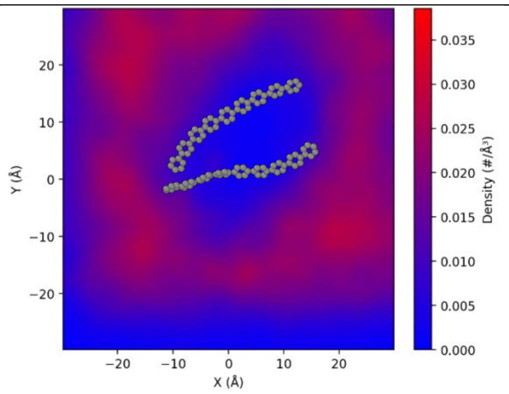	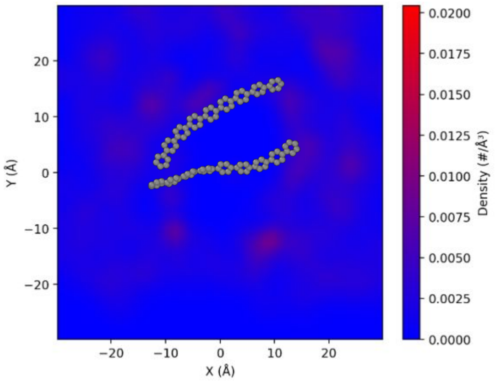

Figure S9. SDF of water and THF around the rods in dimer of C2, C10, and C16 molecules at 33 vol% THF. For better visualization and better understanding that how solvents are distributed around the rods, in each case specific slice in a specific plane of the heatmap has been chosen.

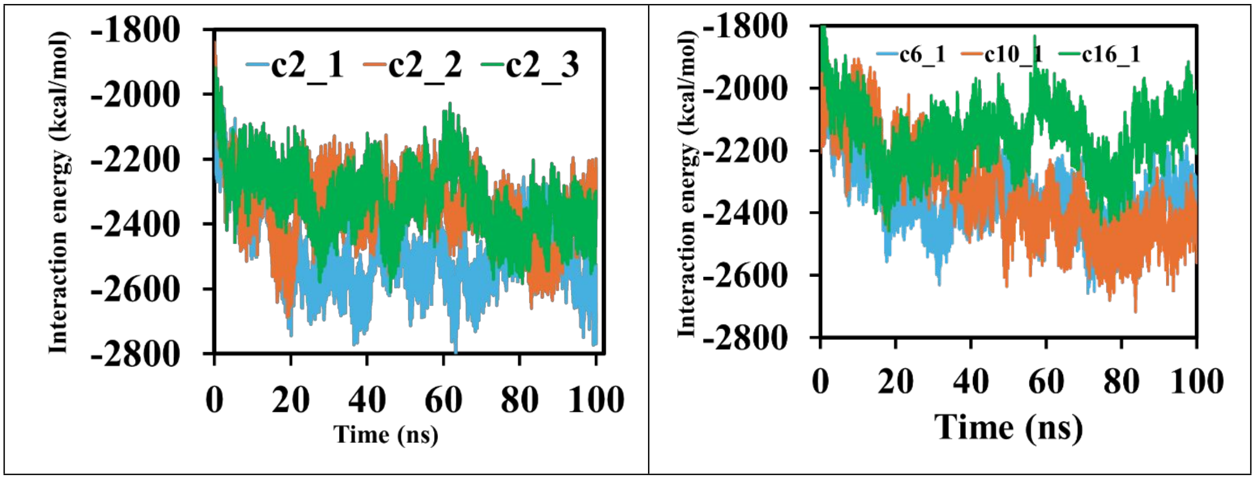

Figure S10. The total interaction energy between the dimers and TBA^+ counterions for 15 vol% THF.

Table S3. Time average interaction energies between the amphiphilic molecules and TBA^+ counterions after dimer formation at 33 vol% THF.

**Molecule type**	C2			C6			C10			C16		
**Simulation number**	1st	2nd	3rd	1st	2nd	3rd	1st	2nd	3rd	1st	2nd	3rd
**Electrostatic (kcal/mol)**	-1776 ± 58	-1995 ± 63	-1842 ± 61	-1863 ± 65	-1930 ± 60	-1826 ± 57	-1914 ± 43	-1718 ± 60	-1946 ± 66	-1865 ± 70	-1836 ± 69	-1788 ± 64
**van der Waals (kcal/mol)**	-61 ± 14	-115 ± 10	-77 ± 13	-84 ± 17	-110 ± 8	-79 ± 11	-105 ± 10	-59 ± 10	-82 ± 10	-74 ± 11	-76 ± 18	-67 ± 11
**Total (kcal/mol)**	-1837 ± 63	-2071± 70	-1918 ± 72	-1947 ± 77	-2041 ± 65	-1905 ± 64	-1956 ± 76	-1777 ± 64	-1876 ± 100	-1939 ± 76	-1912 ± 80	-1856 ± 71

Table S4. The average distance between the center of TBA^+ counterion and the center of the Keggin at 33 vol% THF.

	**C2**	**C6**	**C10**	**C16**
**1**	8.5 ± 0.6	8.8 ± 0.5	8.4 ± 0.6	8.4 ± 0.5
**2**	8.2 ± 0.3	8.7 ± 0.6	8.5 ± 0.3	8.4 ± 0.3
**3**	8.6 ± 0.6	8.5 ± 0.5	8.7 ± 0.6	8.5 ± 0.7

Table S5. The total energy of the 12 different simulations at 33 vol% THF. All the system's components (RSRA, counterion, and solvents) are included for calculating the total energy of each system.

	**Total energy of the systems (kcal/mol)**		
	1st run	2nd run	3rd run
C2	-12565.28 ± 155.84	-12547.42 ± 155.69	-12568.91 ± 165.66
C6	-11936.42 ± 154.22	-11963.63 ± 156.96	-11894 ± 157.12
C10	-11816.29 ± 159.69	-11713.82 ± 157.42	-11656.77 ± 158.25
C16	-11274.64 ± 154.89	-11358.95 ± 159.22	-11231.98 ± 161.59

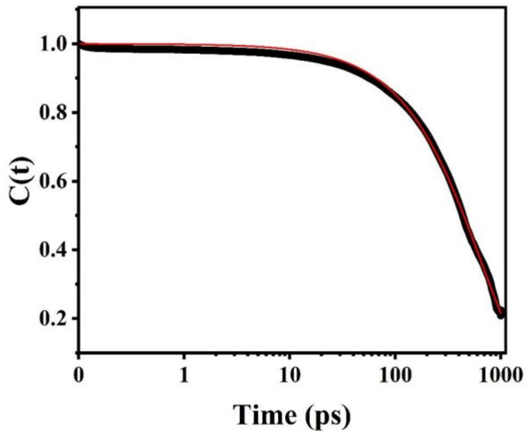

Figure S11. Auto-Correlation function of residence time of water molecules around the Keggin, the data points are in black, and the fitted curve is shown in red. The data for only C6-3 is reported, because of the behavior of the water

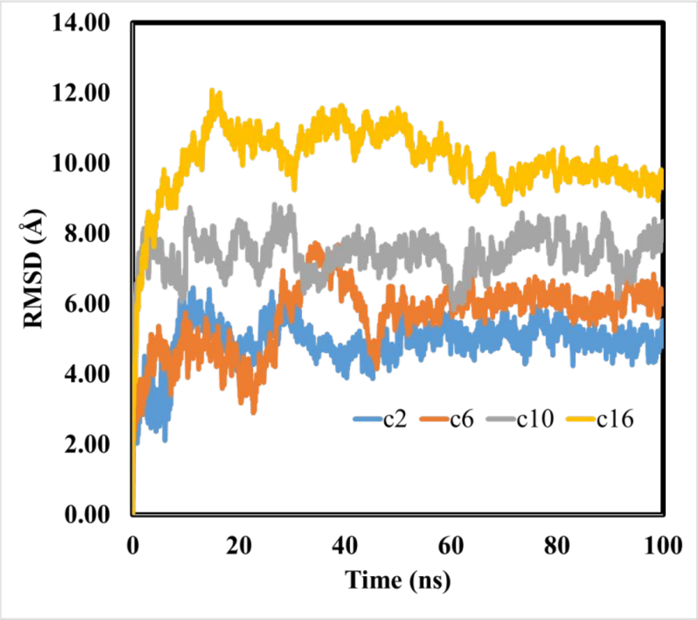

Figure S12. Root-mean-square deviation (RMSD) of a single RSRA as a function of simulation time for systems at 33 vol% THF. RMSD was calculated for one RSRA in each simulation with respect to its configuration at the beginning of the production run and is used to monitor the structural evolution of the molecule during and after dimer formation. The different curves correspond to the four side-chain lengths (C2, C6, C10, and C16). The RMSD behavior observed for the 15 vol% THF systems is qualitatively similar and therefore not shown.

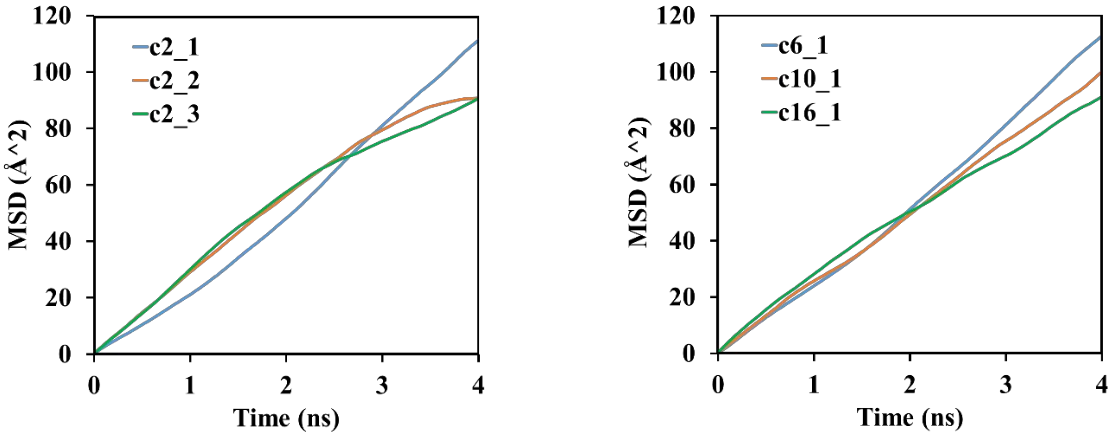

Self-diffusion coefficient ($cm^2/s \times 10^{-7}$)	
C2_1	4.41
C2_2	4.33
C2_3	4.21
C6_1	4.50
C10_1	4.16
C16_1	3.98

Figure S13. Comparison of MSD and self-diffusion coefficient of different simulations in 15 vol% THF.
